# Design of Diaphragm and Coil for Stable Performance of an Eddy Current Type Pressure Sensor

**DOI:** 10.3390/s16071025

**Published:** 2016-07-01

**Authors:** Hyo Ryeol Lee, Gil Seung Lee, Hwa Young Kim, Jung Hwan Ahn

**Affiliations:** 1 School of Mechanical Engineering, Pusan National University, 2, Busandaehak-ro 63 beon gil, Geumjeong-gu, Busan 46241, Korea; hong30140@pusan.ac.kr; 2 NOVASEN Co., LTD, 25, Bansong-ro 525 beon gil, Haeundae-gu, Busan 48002, Korea; novasen@naver.com; 3 Research Institute of Mechanical Technology, Pusan National University, 2, Busandaehak-ro 63 beon gil, Geumjeong-gu, Busan 46241, Korea; hyokim@pusan.ac.kr

**Keywords:** eddy current, pressure sensor, round-groove, impedance change

## Abstract

The aim of this work was to develop an eddy current type pressure sensor and investigate its fundamental characteristics affected by the mechanical and electrical design parameters of sensor. The sensor has two key components, i.e., diaphragm and coil. On the condition that the outer diameter of sensor is 10 mm, two key parts should be designed so as to keep a good linearity and sensitivity. Experiments showed that aluminum is the best target material for eddy current detection. A round-grooved diaphragm is suggested in order to measure more precisely its deflection caused by applied pressures. The design parameters of a round-grooved diaphragm can be selected depending on the measuring requirements. A developed pressure sensor with diaphragm of t = 0.2 mm and w = 1.05 mm was verified to measure pressure up to 10 MPa with very good linearity and errors of less than 0.16%.

## 1. Introduction

Pressure sensors have been widely used in such areas as chemical plants, automobile/ship, aviation, air conditioning, biomedical, and hydraulics industries, etc., where pressure is an important monitored parameter. In chemical plants, pressure sensors are used to monitor partial pressures of gases in industrial units so that large chemical reactions take place under precisely controlled environmental conditions. In automobile and ship engines where it forms an integral part of the engine and its safety, the dynamic cylinder pressure in the combustion chamber is monitored to regulate the power that the engine must deliver to achieve suitable speeds. Engine combustion pressure analysis is a fundamental measurement technique applied universally in the research and development of reciprocating combustion engines [1,2].

The three types of pressure sensors—metal thin-film, ceramic thick-film and piezoresistive—have been most commonly used so far. Among them, silicon piezoresistive MEMS pressure sensors as micromachined devices, are widely used in various systems because of their high accuracy, high sensitivity, and excellent linearity over a wide range of pressures in addition to their low cost and small size [3,4,5,6,7]. Pressure sensors are often exposed to harsh environments with contaminants—such as dust, dirt, and oil—and severe temperature changes, which cause the silicon based sensors to have poorer accuracy, sensitivity, and linearity.

In an effort to develop a robust pressure sensor that can endure such harsh environments, an eddy current type pressure sensor is proposed in this research, due to the high resolution, high frequency response, and robustness against contaminants of an eddy current sensor (ECS). ECS are known to be very competitive for displacement measurement in harsh environments under dynamic and static pressures. Furthermore, active temperature compensation to reduce the temperature effect on ECS has been proved possible through previous research [8,9].

An eddy current type pressure sensor should be composed of two transducer parts—a diaphragm to convert pressure into diaphragm displacement and an eddy current detector to convert diaphragm displacement into eddy current voltage. In this paper, particularly, it is described how the design parameters of these two key components affect the pressure sensor performance—i.e., pressure range and sensitivity—and what type of diaphragm measure the eddy current sensor displacement most stably and precisely. Finally, the characteristic of an assembled sensor of diaphragm and eddy current probe was tested in the real pressure chamber and the feasibility for practical use was checked.

## 2. Principle and Structure of Eddy Current Type Pressure Sensor

### 2.1. Working Principle of a Typical Pressure Sensor

A typical—generally a force collector type—pressure sensor is composed of a diaphragm and a probe, where a pressurized gas from a high pressure chamber causes the diaphragm to be elastically deflected. And the probe then detects the diaphragm deflection. According to detection principles, there are several kinds of pressure sensors such as strain gauge type, piezoresistive type, piezoelectric type, capacitive type, electromagnetic type, and optical type. In strain gauge, piezoresistive, and piezoelectric types, the unit that detects the diaphragm deflection is connected to or built into the diaphragm as a thin layer-bonded, and sputtered etc., while the others are separately located near the diaphragm to detect its deflection in non-contact mode. Piezoresistive sensors—based on measurement of the diaphragm displacement—have a good feature in that the resistance change for a given amount of pressure is much greater than in metal strain gauges.

The eddy current type pressure sensor which is to be developed in this research is one of the electromagnetic type with the diaphragm deflection being detected by a sensor coil. 

### 2.2. Structure of Eddy Current Type Pressure Sensor

**Figure 1:**
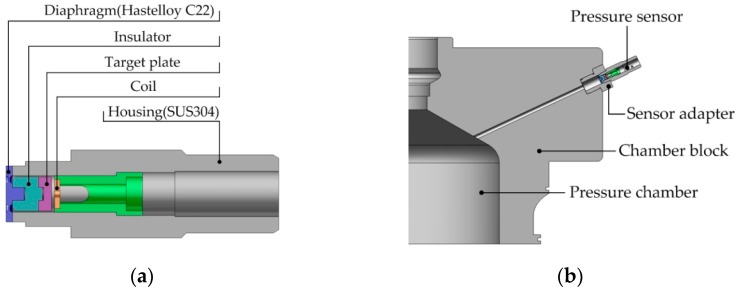
Eddy current type pressure sensor: (**a**) structure; (**b**) on site application.

The structure of an eddy current type pressure sensor to be developed in this study is shown in Figure 1a. The sensor is composed of housing, diaphragm, insulator, target plate and sensor coil (eddy current probe). The diaphragm is made of Hastelloy C22 to prevent sensors from being rusted even under harsh conditions. When pressurized gas from a high pressure chamber is applied to the sensor, as shown in Figure 1b, the diaphragm is deflected, which moves the insulator and the target plate; the resulting displacement of the target plate is then detected by the coil. 

Therefore, the two key parts of an eddy current type pressure sensor—diaphragm and coil—should be well-matched electrically and mechanically to enhance its sensitivity and linearity in the aspect of tiny displacement acquisition on the condition that the diaphragm deflection is within the elastic limit. For compatible use with other types of commercialized pressure sensors, the diameter of the sensor is fixed at 10 mm.

## 3. Design of Sensor Coil

### 3.1. Design Parameters

**Figure 2:**
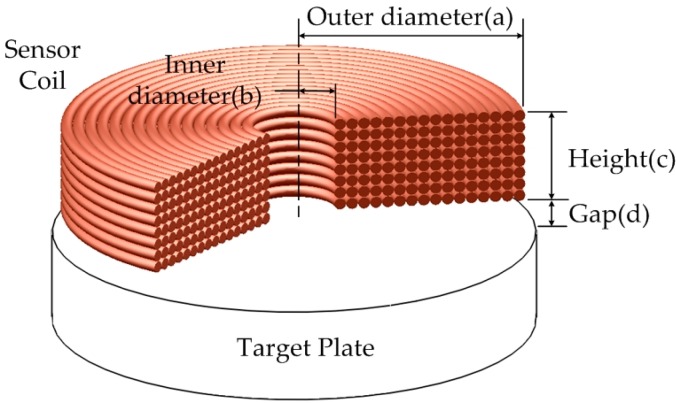
Geometry of coil against target plate.

Figure 2 shows a schematic diagram of the sensor coil unit—eddy current probe. 

Many design parameters are geometric ones, such as: outer diameter (a); inner diameter (b); and height (c) of the coil unit; coil diameter and gap (d) between coil and target plate. Other design parameters include magneto-electrical ones such as conductivity, temperature resistance and magnetic permeability. It is known that self-inductance and mutual-inductance of the sensor coil are dependent only on geometrical parameters [10]. Copper is used as coil material to get sensitivity and linearity of detection as high as possible because of its high conductivity and low temperature resistance. 

From the viewpoint of sensor performance referring to the past works, the inner and outer diameter should be minimized and maximized, respectively, and the height should be minimized as much as geometrically possible [10,11]. Since the coil should detect the diaphragm deflection appropriately, it is desirable that the diameter of the probe is limited to a maximum of 5 mm. The inner diameter and height should be at least 2 mm and 1 mm respectively for convenience of coil manufacturing. The copper wire diameter is at most 0.1 mm, as anything larger reduces the sensitivity and the anything smaller is too sensitive to disturbance. The gap is fixed at 0.3 mm to avoid the effect of parasitic capacitance largely generated in ranges less than 0.2 mm.

### 3.2. Signal Processing for Eddy Current Detection

**Figure 3:**
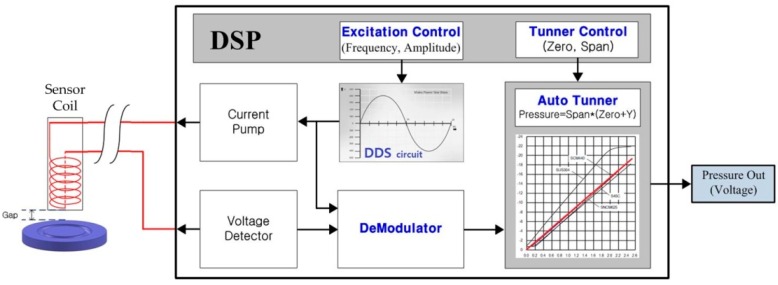
Functional block diagram for signal processing of eddy current acquisition.

The principle of eddy current detection is to detect a variation of coil impedance caused by a change of the gap between coil and target plate. Figure 3 shows a signal processing block for detecting a change of coil impedance and removing disturbances from the acquired R and L impedances.

Given the optimal frequency (250 kHz) and amplitude of the coil excitation current (25 mA) by DSP (Digital Signal Processor), a DDS (Direct Digital Synthesizer) circuit transmits a corresponding voltage signal to a current pump which supplies a corresponding current to the coil. Eddy current electromagnetically induced in the target plate placed close to the sensor coil causes a change of voltage in the sensor coil, which is detected by a voltage detector and is converted to a change of the coil impedance by a demodulator circuit.

### 3.3. Experiment Evaluation of Sensor Coil

**Figure 4:**
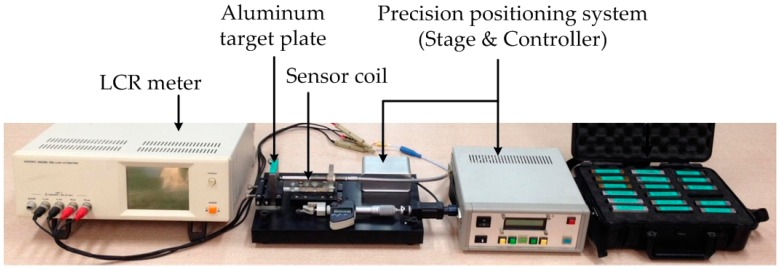
Coil impedance measurement system with gap controlled.

The characteristics of the sensor coil are examined along with the gap precisely controlled on a measurement system, as shown in Figure 4, which is composed of a precision positioning unit controlling the gap and an LCR (inductance (L), capacitance (C), and resistance (R)) meter measuring coil impedance.

**Figure 5:**
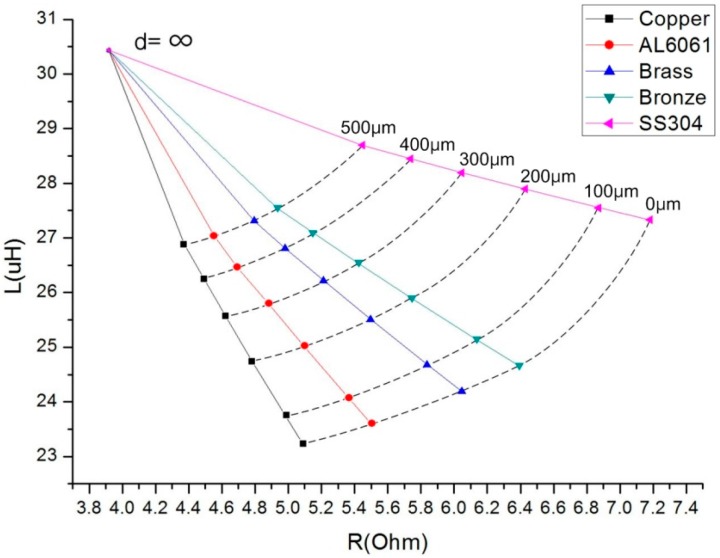
Coil impedance measurement system with gap controlled.

In order to find out the best target material in the magneto electrical aspect, five materials—copper, aluminum, brass, bronze and stainless steel—are considered as alternatives. The results of impedance measurements for the five target materials are displayed in the R-L impedance plane, which is specially adopted to improve the typical measurement method based on amplitude data acquired from an LCR meter, as shown in Figure 5. 

Both impedance changes against gap—ΔL/Δd, ΔR/Δd—are almost linear in a range less than 500 μm, although they become nonlinear as the gap increases beyond that. As R is actually affected more than L by temperature change, copper or aluminum is preferred over the other materials as the target material, because of higher conductivity and lower temperature sensitivity in addition to nonmagnetic metals property. Furthermore, in terms of corrosion-resistance, aluminum is much better than copper. Therefore, Al6061 is chosen as the target material.

## 4. Design of Round-Grooved Diaphragm

### 4.1. Shape of Diaphragm for Stably Measurable Deflection

The diaphragm plays an important role in the elastic deflection in response to an applied pressure. The shape and geometrical dimensions of a diaphragm should be well-chosen so that its deflection matches the performance of the sensor coil designed in Section 3. A small sensor diameter of 10 mm limits the diaphragm diameter up to 8.6 mm at most. Furthermore, its deflection being measured more accurately by the sensor coil, the diaphragm should be designed so that some area of its central part goes up and down near flatly as the acting pressure goes up or down. 

**Figure 6:**
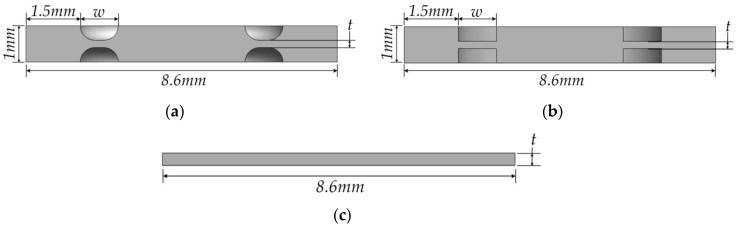
Shape and design parameter of three types of diaphragm: (**a**) round-grooved; (**b**) square grooved; (**c**) flat.

What shape of the diaphragm is needed to meet such restricted requirements? Wang et al. revealed that the center-embossed diaphragm is superior in optical characteristics to the flat one which is seriously deteriorated by the central area being wrapped under high hydrostatic pressure [12]. In this study, a round-grooved, square-grooved diaphragm—like a center-embossed one—shown in Figure 6a,b, are considered for rather flatter deflection of the central part, which helps the eddy current probe measure a gap more stably and precisely. The design parameters, as depicted in Figure 6a,b, are the width and thickness of the groove while the thickness of the diaphragm and the distance of the groove from the wall are set as 1 mm and 1.5 mm, respectively. For deflection comparison a flat type, shown in Figure 6c, is used as a reference.

### 4.2. Analysis of Diaphragm Deflection

Structural analyses were carried out to investigate the effects of design parameters on the maximum deflection and stress of the diaphragm by increasing pressure in 0.25 MPa increments until yielding occurs using ANSYS Workbench V15. The material properties of Hastelloy C22 are elastic modulus 206 GPa, yield strength 438 MPa, and Poisson ratio 0.3.

**Figure 7:**
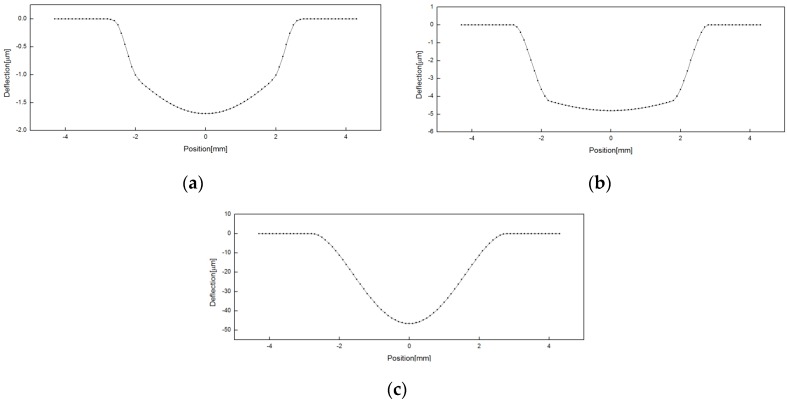
Deflection behavior along the diaphragm axis with t = 0.2 mm, w = 1.05 mm, P = 2.5 MPa: (**a**) round-grooved; (**b**) square-grooved; (**c**) flat.

With thickness (t) 0.2 mm and pressure 2.5 MPa, Figure 7 shows a typical deflection behavior along the radial axis of the round-grooved, square-grooved, and flat diaphragms, respectively. 

The point 0 indicates the center of the diaphragm. The deflection of the whole diaphragm surface under 2.5 MPa is displayed on the axis of −4.3~4.3 mm. While a much larger deflection with no flat central part is seen in the flat type, the grooved type deflects much less with quite a flat deflection in the center part compared to the flat type, which makes the eddy current probe measure the diaphragm deflection with more stability and precision. 

**Figure 8:**
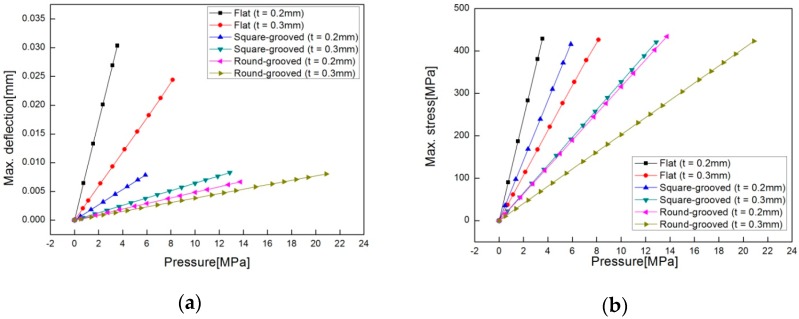
Comparison of maximum deflection/stress against pressure among flat, square-grooved, and round-grooved types with w = 1.05 mm: (**a**) maximum deflection; (**b**) maximum stress.

Figure 8 shows comparisons of the simulated results in terms of maximum deflection and maximum stress at each pressure between flat, square-grooved, and round-grooved types with increasing pressure at the same thickness.

**Figure 9:**
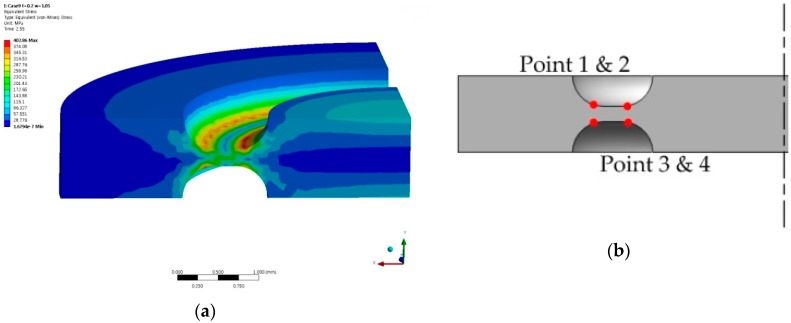
Analysis results at t = 0.2 mm, w = 1.05 mm, P = 12.5 MPa: (**a**) stress distribution; (**b**) yield points.

For t = 0.2 mm, the flat type has a yielding pressure around at 3.55 MPa, which is much lower than the round-grooved type at 13.75 MPa or the square-grooved type at 5.87 MPa. For t = 0.3 mm, the flat type has a yielding pressure around 8.15 MPa, which is much lower than the round-grooved type at 20.88 MPa or square-grooved type at 12.87 MPa. Figure 9 shows the numerical analytical results of how the stress is distributed on the round-grooved diaphragm and how the yielding stress occurs at points 1, 2, 3, and 4 on both surfaces of the round-groove with t = 0.2 mm at 12.5 MPa.

The round-grooved type has a wider pressure range linearly measurable prior to yielding than the square-grooved and the flat types. Thus, only the round-grooved type is considered for further investigation of the effects of the design parameters.

Table 1 summarizes the 16 analysis cases with the two design parameters properly selected for the round-grooved diaphragm and their numerical analytical results of maximum pressure and deflection. In addition, the sensitivity is calculated as maximum deflection/maximum pressure.

**Figure 10:**
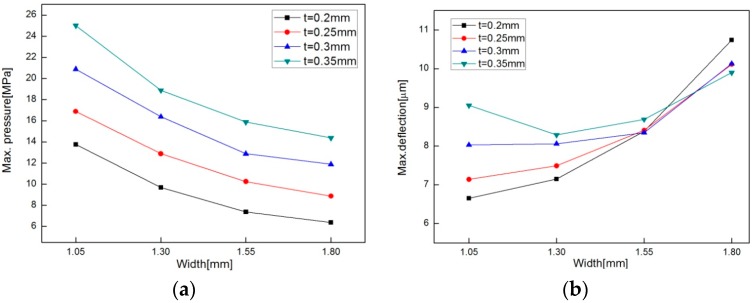
Maximum pressure (**a**) and maximum deflection (**b**) with design parameters of round-groove type.

Figure 10 shows the maximum pressure and deflection at varied thickness and width. Figure 10a indicates that as the round-groove thickness increases and width decreases, the maximum pressure prior to yielding becomes larger. The change rates affected by both parameters are almost equal. Figure 10b shows that the round-groove width affects the deflection much more than the round-groove thickness but does not show a consistent trend when thickness increased. So it would be better to use the sensitivity calculated in Table 1 as an indicator to compare how sensitive the diaphragm is to pressure. The thinner and wider one is more sensitive to pressure change. The larger width increases the sensitivity much more than the thickness but lowers the measurable maximum pressure too much, as shown in Figure 10a.

Based on the measurement requirements of pressure and sensitivity, the design parameters of diaphragm can be selected. In this study the round-groove diaphragm with a groove 0.2 mm thick and 1.05 mm wide is chosen to investigate its performances throughout the experiment.

## 5. Sensor Performance Evaluation through Pressure Measurement Test

### 5.1. Measurements of Diaphragm Deflection According to Applied Pressure

**Figure 11:**
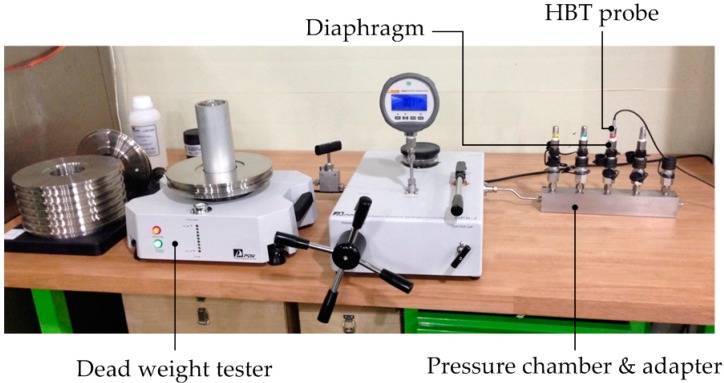
Diaphragm deflection measurement equipment with pressure varied by dead weights.

It is necessary to examine how well the diaphragm deflection relates to the applied pressure. Figure 11 shows a setup of an apparatus composed of a pressure chamber, dead weight tester and half bridge transformer (HBT) for the experiment.

**Figure 12:**
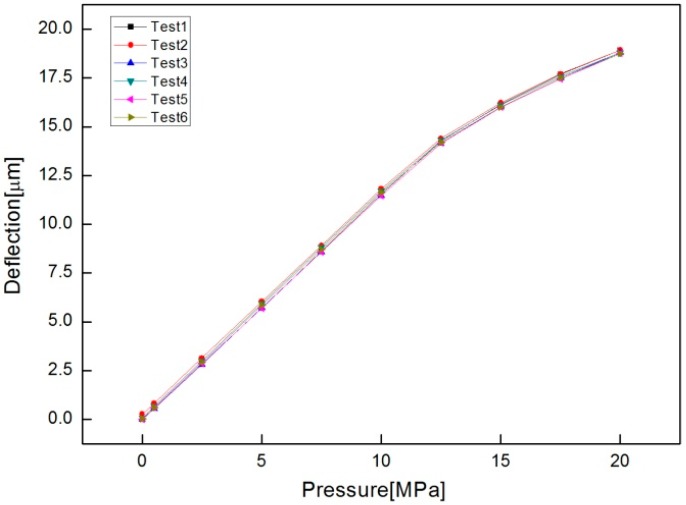
Plot of measured diaphragm deflection with pressure.

The deflection is measured by a HBT with pressure increased by 2.5 MPa increments up to 20 MPa. To evaluate the repeatability of diaphragm deflection in response to changes in pressure, the measurement experiments were performed six times with a single specimen. The measurement results are listed in Table 2 and displayed in Figure 12, which shows almost the same behavior.

The deflection-pressure curve appears linear up to about 12.5 MPa lower than the simulated yielding pressure of 13.75 MPa and the elasticity seems to remain even up to 20 MPa, although the deflection rate against pressure becomes lower. This would be due to work hardening of the round-groove of the diaphragm. Through regression analysis the linear relationship between diaphragm deflection (δ) and pressure (P), δ (μm) = 1.142P (MPa) + 0.087, is of such good fitness that a coefficient of determination of 0.999 is obtained.

### 5.2. Assembled Pressure Sensor

**Figure 13:**
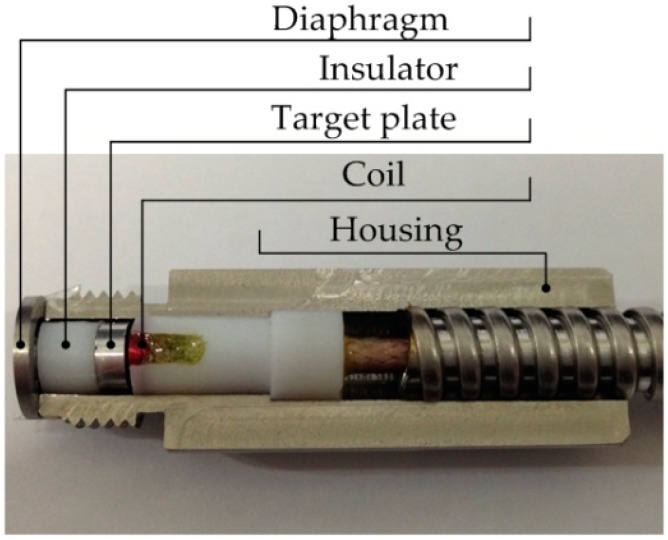
Cross section view of an assembled pressure sensor.

Figure 13 shows a cross section view of an assembled pressure sensor developed for this research. 

Its round-groove machined in both faces by turning, a round-grooved diaphragm was welded on the one end of housing. An insulator was placed between diaphragm and target plate to prevent heat transfer from the diaphragm since the coil impedance is affected by temperature. 

### 5.3. Performance Evaluation of an Assembled Pressure Sensor According to Applied Pressure

**Figure 14:**
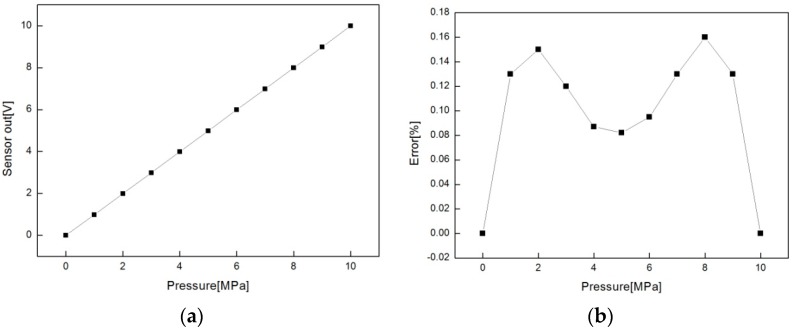
Pressure sensor output (**a**) and linearity error (**b**) along with applied pressure

The performance of an assembled sensor was tested on the same equipment as in Figure 11 with a HBT probe replaced by a multi-meter to measure the pressure output (voltage) depicted in Figure 3. The measurement results are summarized in Table 3 and the sensor output is displayed in Figure 14a. The sensor output is linearly well related with input pressure up to 10 MPa and has maximum linearity errors less than 0.16%, as seen in Figure 14b, which is much smaller than other commercialized sensors.

## 6. Conclusions

An eddy current type pressure sensor with an outer diameter of 10 mm was developed and its characteristic was investigated so as to get appropriate values of the design parameters of the sensor’s two key components, i.e., diaphragm and coil. Some important findings are summarized as follows.

(1) The coil designed in this research has a good linear relationship between the impedance change and the distance to target plate up to 500 μm.
(2) A round-grooved diaphragm is much better for stable measurement of deflection even with much less deflection compared to a flat type one.
(3) Depending on the required pressure and sensitivity measurement, the design parameters of a round-grooved diaphragm can be selected.
(4) The developed sensor shows a very good linearity up to 10 MPa with linearity errors less than 0.16%.

## Figures and Tables

**Table 1:** 16 cases of properly selected design parameters with analytical results of maximum pressure, maximum deflection and sensitivity.

	Thichness (mm)	Width (mm)	Max. Pressure (MPa)	Max. Deflection (μm)	Sensitivity (μm/MPa)
Case 1	0.20	1.05	13.75	6.65	0.48
Case 2	0.20	1.30	9.68	7.15	0.74
Case 3	0.20	1.55	7.38	8.38	1.14
Case 4	0.20	1.80	6.38	10.74	1.68
Case 5	0.25	1.05	16.88	7.14	0.42
Case 6	0.25	1.30	12.88	7.49	0.58
Case 7	0.25	1.55	10.25	8.41	0.82
Case 8	0.25	1.80	8.88	10.11	1.14
Case 9	0.30	1.05	20.88	8.03	0.38
Case 10	0.30	1.30	16.38	8.06	0.49
Case 11	0.30	1.55	12.88	8.35	0.65
Case 12	0.30	1.80	11.88	10.13	0.82
Case 13	0.35	1.05	25.00	9.05	0.36
Case 14	0.35	1.30	18.88	8.29	0.44
Case 15	0.35	1.55	15.88	8.69	0.55
Case 16	0.35	1.80	14.38	9.90	0.69

**Table 2:** 6 times measured results of diaphragm deflection according to varied pressure

Pressure (MPa)	Test 1 (μm)	Test 2 (μm)	Test 3 (μm)	Test 4 (μm)	Test 5 (μm)	Test 6 (μm)	Average	Standard Deviation
0.0	0.00	0.26	0.00	0.09	0.00	0.02	0.06	0.10
0.5	0.57	0.82	0.56	0.68	0.59	0.61	0.64	0.10
2.5	2.85	3.14	2.82	3.00	2.85	2.93	2.93	0.12
5.0	5.70	6.05	5.72	5.94	5.70	5.85	5.83	0.15
7.5	8.60	8.90	8.59	8.82	8.58	8.72	8.70	0.14
10.0	11.55	11.81	11.47	11.7	11.48	11.63	11.61	0.13
12.5	14.27	14.4	14.15	14.3	14.16	14.21	14.25	0.10
15.0	16.14	16.22	16.01	16.11	16.00	16.02	16.08	0.09
17.5	17.69	17.71	17.50	17.58	17.47	17.50	17.57	0.11
20.0	18.94	18.94	18.80	18.8	18.76	18.76	18.83	0.09

**Table 3:** Measurement results of the assembled sensor according to varied pressure.

Pressure (MPa)	Impedance Change (mΩ)	Sensor Output (V)	Error (%)
0	0.00	0.00	0.00
1	11.66	0.98	0.13
2	23.45	1.99	0.15
3	35.29	2.99	0.12
4	47.14	3.99	0.09
5	58.96	4.99	0.08
6	70.75	5.99	0.10
7	82.52	6.99	0.13
8	94.30	7.99	0.16
9	106.14	8.99	0.13
10	118.10	10.00	0.00

* Error (%) = (Sensor output (V)—Expected value(V))/(10V) × 100.
